# New developments on the cheminformatics open workflow environment CDK-Taverna

**DOI:** 10.1186/1758-2946-3-54

**Published:** 2011-12-13

**Authors:** Andreas Truszkowski, Kalai Vanii Jayaseelan, Stefan Neumann, Egon L Willighagen, Achim Zielesny, Christoph Steinbeck

**Affiliations:** 1 Institute for Bioinformatics and Cheminformatics, University of Applied Sciences of Gelsenkirchen, Recklinghausen, Germany; 2 Chemoinformatics and Metabolism, European Bioinformatics Institute (EBI), Cambridge, UK; 3 GNWI - Gesellschaft fuer naturwissenschaftliche Informatik mbH, Oer-Erkenschwick, Germany; 4 Division of Molecular Toxicology, Institute of Environmental Medicine, Karolinska Institutet, Stockholm, Sweden

## Abstract

**Background:**

The computational processing and analysis of small molecules is at heart of cheminformatics and structural bioinformatics and their application in e.g. metabolomics or drug discovery. Pipelining or workflow tools allow for the Lego™-like, graphical assembly of I/O modules and algorithms into a complex workflow which can be easily deployed, modified and tested without the hassle of implementing it into a monolithic application. The CDK-Taverna project aims at building a free open-source cheminformatics pipelining solution through combination of different open-source projects such as Taverna, the Chemistry Development Kit (CDK) or the Waikato Environment for Knowledge Analysis (WEKA). A first integrated version 1.0 of CDK-Taverna was recently released to the public.

**Results:**

The CDK-Taverna project was migrated to the most up-to-date versions of its foundational software libraries with a complete re-engineering of its worker's architecture (version 2.0). 64-bit computing and multi-core usage by paralleled threads are now supported to allow for fast in-memory processing and analysis of large sets of molecules. Earlier deficiencies like workarounds for iterative data reading are removed. The combinatorial chemistry related reaction enumeration features are considerably enhanced. Additional functionality for calculating a natural product likeness score for small molecules is implemented to identify possible drug candidates. Finally the data analysis capabilities are extended with new workers that provide access to the open-source WEKA library for clustering and machine learning as well as training and test set partitioning. The new features are outlined with usage scenarios.

**Conclusions:**

CDK-Taverna 2.0 as an open-source cheminformatics workflow solution matured to become a freely available and increasingly powerful tool for the biosciences. The combination of the new CDK-Taverna worker family with the already available workflows developed by a lively Taverna community and published on myexperiment.org enables molecular scientists to quickly calculate, process and analyse molecular data as typically found in e.g. today's systems biology scenarios.

## Background

Current problems in the biosciences typically involve several domains of research. They require a scientist to work with different and diverse sets of data. The reconstruction of a metabolic network from sequencing data, for example, employs many of the data types found along the axis of the central dogma, including reconstruction of genome sequences, gene prediction, determination of encoded protein families, and from there to the substrates of enzymes, which then form the metabolic network. In order to work with such a processing pipeline, a scientist has to copy/paste and often transform the data between several bioinformatics web portals by hand. The manual approach involves repetitive tasks and cannot be considered effective or scalable.

Especially the processing and analysis of small molecules comprises tasks like filtering, transformation, curation or migration of chemical data, information retrieval with substructures, reactions, or pharmacophores as well as the analysis of molecular data with statistics, clustering or machine learning to support chemical diversity requirements or to generate quantitative structure activity/property relationships (QSAR/QSPR models). These processing and analysis procedures itself are of increasing importance for research areas like metabolomics or drug discovery. The power and flexibility of the corresponding computational tools become essential success factors for the whole research process.

The workflow paradigm addresses the above issues with the supply of sets of elementary workers (activities) that can be flexibly assembled in a graphical manner to allow complex procedures to be performed in an effective manner - without the need of specific code development or software programming skills. Scientific workflows allow the combination of a wide spectrum of algorithms and resources in a single workspace [1-3]. Earlier problems with iterations over large data sets [4] are completely resolved in version 2.0 due to new implementations in Taverna. Taverna 2 allows control structures such as "while" loops or "if-then-else" constructs. Termination criteria for loops may now be evaluated by listening to a state port [5]. In addition the user interface of the Taverna 2 workbench has clearly improved: The design and manipulation of workflows in a graphical workflow editor is now supported. Features like copy/paste and undo/redo simplify workflow creation and maintenance [6].

The CDK-Taverna project aims at building a free open-source cheminformatics pipelining solution through combination of different open-source projects such as Taverna [7], the Chemistry Development Kit (CDK) [8,9], or the Waikato Environment for Knowledge Analysis (WEKA) [10]. A first integrated version 1.0 of CDK-Taverna was recently released to the public [4]. To extend usability and power of CDK-Taverna for different molecular research purposes the development of version 2.0 was motivated.

### Implementation

The CDK-Taverna 2.0 plug-in makes use of the Taverna plug-in manager for its installation. The manager fetches all necessary information about the plug-in from a XML file which is located at http://www.ts-concepts.de/cdk-taverna2/plugin/. The information provided therein contains the name of the plug-in, its version, the repository location and the required Taverna version. Upon submitting the URL to the plug-in manager it downloads all necessary dependencies automatically from the web. After a subsequent restart the plug-in is enabled and the workers are visible in the services. The plug-in uses Taverna version 2.2.1 [6], CDK version 1.3.8 [11] and WEKA version 3.6.4 [12]. Like its predecessor it uses the Maven 2 build system [13] as well as the Taverna workbench for automated dependency management.

### CDK-Taverna 2.0 worker implementation

The CDK-Taverna 2.0 plug-in is designed to be easily extendible: The implementation allows to create new workers by simply inheriting from the single abstract class org.openscience.cdk.applications.taverna.AbstractCDKActivity (which is the analogue of the CDKLocalWorker interface of CDK-Taverna version 1.0). The class is located in the cdk-taverna-2-activity module. It provides all necessary data for the underlying worker registration mechanism which frees the software developer from handling these tasks manually. The methods which need to be overwritten in order to implement a worker are:

• public void addInputPorts(), public void addOutputPorts(): Specify the ports for passing data between workers.

• public String getActivityName(), public String getFolderName(): Return name and folder of a worker.

• public void work(): Entry point for the worker's central algorithm that performs its core function.

• public String getDescription(): Provides descriptive text that explains a worker's function.

• public HashMap <String, Object> getAdditionalProperties(): Specifies additional properties like file extensions, the number of concurrent threads to use, etc.

Finally a new worker has to be registered to be available in the Taverna workbench. For this purpose Taverna offers the class net.sf.taverna.t2.spi.SPIRegistry.SPIRegistry to register Service Provider Interfaces (SPI). It is necessary to add the new worker's full name including its package declaration to the file org.openscience.cdk.applications.taverna.AbstractCDKActivity which contains the names and packages of all available workers. This file is located at cdk-taverna-2-activity-ui/src/main/resources/META-INF/services.

Besides the basic implementation it is possible to define a configuration panel for a worker which allows the specification of parameters. A configuration panel has to inherit from the abstract class org.openscience.cdk.applications.taverna.ActivityConfigurationPanel. The GUI element itself has to be defined in the constructor of the class and may contain any Java Swing element. The following methods are the backbone of a configuration panel:

• public boolean checkValues(): Validates all GUI values.

• public boolean isConfigurationChanged(): After the validity check this method is used to compare the current worker settings with the GUI settings to detect changes.

• public void noteConfiguration(): The properties of the worker are saved in a bean structure. The changes of the configuration bean object are updated by this method.

• public void refreshConfiguration(): Updates the GUI values itself.

• public CDKActivityConfigurationBean getConfiguration(): Access to the configuration bean.

The configuration panel has to be registered in the CDKConfigurationPanelFactory class of the org.openscience.cdk.applications.taverna.ui.view package. More details on how to write workers and their configuration panels are provided at the project's wiki page http://cdk-taverna-2.ts-concepts.de/wiki/index.php?title=Main_Page.

### Requirements

CDK-Taverna 2.0 supports 64-bit computing by use with a Java 64-bit virtual machine. The CDK-Taverna 2.0 plug-in is written in Java and requires Java 6 or higher. The latest Java version is available at http://www.java.com/de/download/. The CDK-Taverna 2.0 plug-in is developed and tested on Microsoft Windows 7 as well as Linux and Mac OS/X (32 and 64-bit).

## Results and Discussion

The CDK-Taverna 2.0 plug-in provides 192 workers for input and output (I/O) of various chemical file and line notation formats, substructure filtering, aromaticity detection, atom typing, reaction enumeration, molecular descriptor calculation and data analysis. Parallel computing with multi-core processors by use of multiple concurrent threads is flexibly implemented for many workers where operations scale nearly linear with the number of cores. Especially the machine learning and the molecular descriptor calculation workers benefit from parallel computation. An overview is given in Tables 1 and 2. Many workers are described by example workflows available at http://cdk-taverna-2.ts-concepts.de/wiki/index.php?title=Main_Page. Additionally, the workflows can be found at http://www.myexperiment.org/.

**Table 1:** CDK-Taverna 2.0 workers

Function	# workers	Examples
File I/O	18	SDFReader, SmilesReader
Iterative File I/O	8	IterativeSDFileReader, LoopSDFileReaderActivity
		
String Converter	10	CMLStringToStructureConverter
Molecular descriptor calculation	99	AtomCount, LargestChain, WienerIndex
Machine learning	30	kMeans, Perceptron, SVM
Miscellaneous	27	JChemPaint, ReactionEnumerator

Overview on CDK-Taverna 2.0 workers categorized by their function.

**Table 2:** Overview on multi-threading CDK-Taverna 2

Function	Worker
Calculation of molecular descriptors	QSAR Descriptor Threaded
Significance of input components evaluation using a genetic algorithm	GA Attribute Selection
Significance of input components evaluation using a 'Leave-One-Out' strategy	Leave-One-Out Attribute Selection
Partitioning datasets into training and test sets	Split Dataset Into Train-/Testset
Construction of clustering models	Weka Clustering
Construction of regression models	Weka Regression
Construction of classification models	Weka Classification

Overview on CDK-Taverna 2.0 workers which are capable of using multiple threads for their calculations.

CDK-Taverna 1.0 was confined to 32-bit Java virtual machine and thus was restricted to in-memory processing of data volumes of at most 2 gigabyte in practice. Version 2.0 also supports 64-bit computing by use of a 64-bit Java virtual machine so that the processable data volume is only limited by hardware constraints (memory, speed): 64-bit in-memory workflows were successfully performed with data sets of about 1 million small molecules. Since the memory restrictions of version 1.0 were a main reason to use Pgchem::tigress as a molecular database backend [4] the corresponding version 1.0 workers were not migrated to the current version 2.0 yet.

### Advanced reaction enumeration

**Figure 1:**
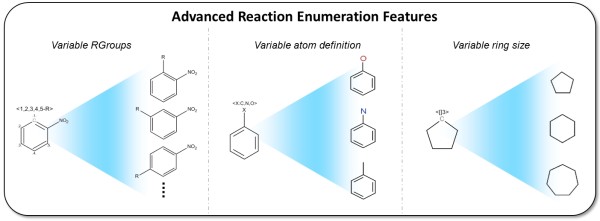
**Advanced reaction enumeration features: (left) The *Variable RGroup *feature allows the definition of chemical groups which can be flexibly attached to predefined atoms**. (middle) The Atom Alias feature offers the possibility to define a wild card for preconfigured elements. (right) The Expandable Atom feature enables the definition of freely sizeable rings or aliphatic chains.

**Figure 2:**
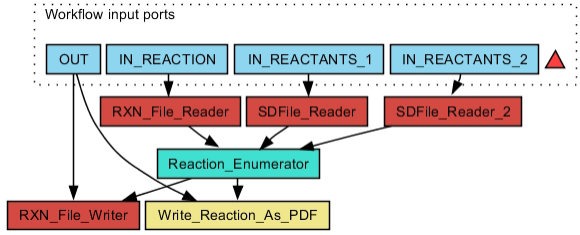
**Workflow for reaction enumeration: After loading a generic reaction (**IN REACTION**, from a MDL RXN file) and two educt lists (**IN REACTANTS 1, IN REACTANTS 2**, from MDL SD files) the **Reaction Enumerator**worker performs the enumeration with the results stored as MDL RXN files**. An additional PDF file is created which shows all enumerated reactions in a tabular manner. The results are stored in the output folder determined by the OUT input port.

**Figure 3:**
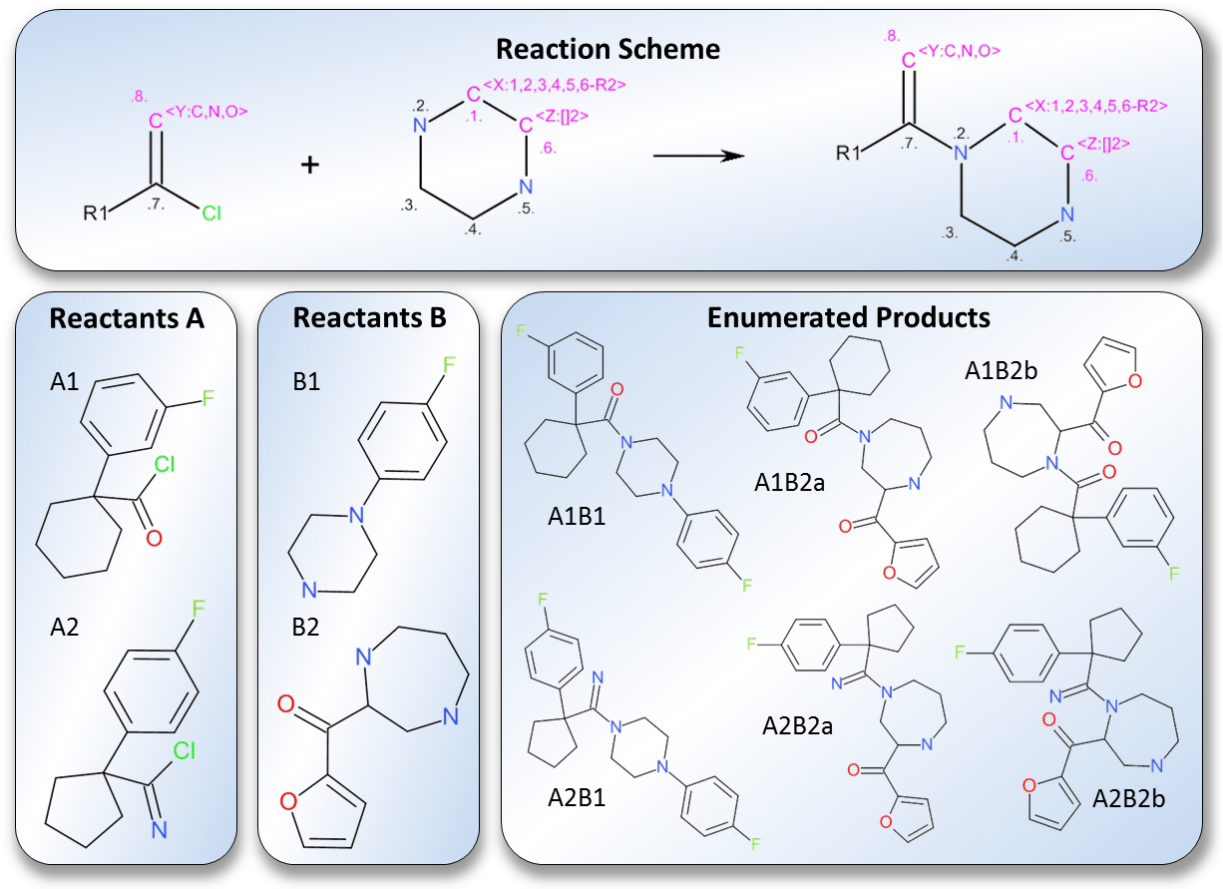
**Capabilities of the advanced reaction enumerator: The sketched generic reaction contains three different generic groups labelled X, Y and Z**. Group × defines a *Variable RGroup *which can freely attach to all atoms of the ring. The *Atom Alias *group labelled Y is a wild card for the elements carbon, oxygen and nitrogen. The *Expandable Atom *group Z defines a variable ring size: The ring can be expanded by up to two additional carbon atoms. The enumerated products with the small letters *a *and *b *originate from *multi-match detection*.

CDK-Taverna 1.0 provided basic functions for combinatorial chemistry related reaction enumeration: They supported the use of two reactants, a single product and one generic group per reactant. The new enumeration options used by CDK-Taverna 2.0 offer major enhancements like multi-match detection, any number of reactants, products or generic groups as well as variable R-groups, ring sizes and atom definitions. The extended functionality was developed and applied in industrial cooperation projects. Advanced reaction enumeration features are illustrated in Figure 1. The *Variable RGroup *feature allows the definition of chemical groups which can be flexibly attached to predefined atoms with syntax *[A:B,B,B...-RC] *where *A *is a freely selectable identifier, *B *are numbers from an *Atom-to-Atom-Mapping *defining the atoms to which the generic group can be attached and *C *is the chemical group identifier which can be any number. The *Atom Alias *feature offers the possibility to define a wild card for preconfigured elements. The syntax is *[A:B,B,B...] *where *A *is a freely selectable identifier and *B *are the string representations of the possible elements. The *Expandable Atom *feature enables the definition of freely sizeable rings or aliphatic chains with syntax *[A:[]B] *where *A *is a freely selectable identifier and *B *is the maximum number of atoms to insert. Figure 2 depicts a workflow for reaction enumeration. The capabilities of the advanced reaction enumerator implementation are summarized in Figure 3 which also demonstrates multi-match detection, i.e. multiple reaction centers within one molecule.

### Evaluation of small molecules for natural product likeness

In recent years, computer assisted drug design studies use natural product (NP) likeness as a criterion to screen compound libraries for potential drug candidates [14,15]. The reason to estimate NP likeness during candidate screening is to facilitate the selection of those compounds that mimic structural features that are naturally evolved to best interact with biological targets.

**Figure 4:**
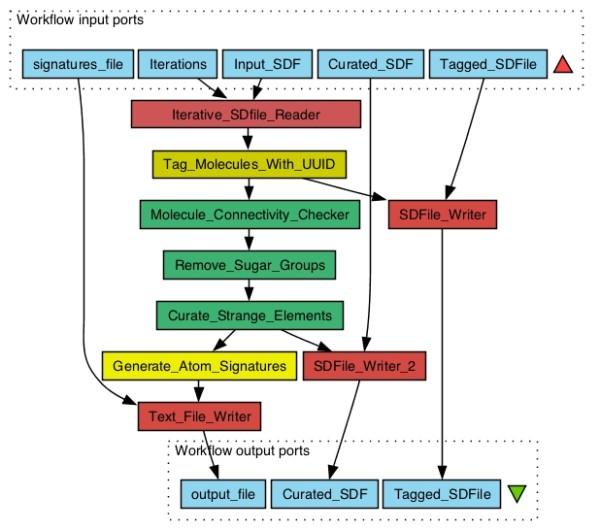
**Molecule curation and atom signature descriptor generation workflow: The **Iterative SDfile Reader**takes the Structure-Data File (SDF) of compounds (**Input SDF**) as input and pass the structures down the workflow for molecule curation and atom signature generation**. The number of structures to be read, and pumped down the workflow can be configured (Iterations). As soon as the molecule is read, the Tag Molecules with UUID worker tags the molecule with Universal Unique IDentifier (UUID) to keep track of it during the process. The Molecule connectivity checker worker checks the connectedness of the structure and removes counter ions and disconnected fragments. The Remove sugar groups worker removes linear and ring sugars from the structures. The Curate Strange Elements worker removes structure containing elements other than non-metals. Finally, the Generate Atom Signatures worker generates atom signature for each atom in a curated compound, tagged with the respective UUID of the compound. The generated atom signatures are written out to a text file (signatures file) using the Text File Writer worker. The SDF of compound structures can be written out to a file, after tagging with UUID (Tagged SDFile), and also after any curation step (Curated SDF) using the SDFile Writer worker. This workflow can be freely downloaded at http://www.myexperiment.org/workflows/2120.html.

**Figure 5:**
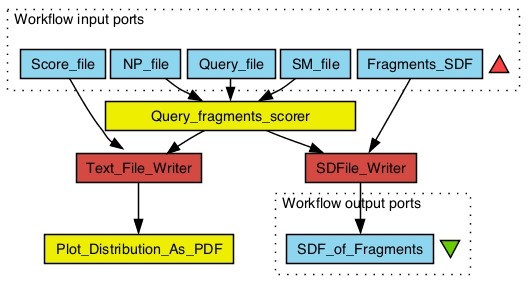
**NP-likeness scoring workflow: This workflow take inputs of atom signatures file generated from the user defined natural products library (**NP file**) as well as synthetics (**SM file**) and compound libraries (**Query file**) and score the compound libraries (**Query file**) for NP-likeness**. The higher the score the more is the NP-likeness of a molecule. The Query fragments scorer worker generates score for each compound in the Query file tagged with the corresponding UUID of the compound. Pairs of compound's UUID and score are written out to a text file (Score file) which can also be passed to the Plot Distribution As PDF worker to see the distribution of the score density of the complete query dataset. The Query fragments scorer worker also regenerates structure for every atom signature and tags it with its corresponding fragment score and UUID of the compound to which it belong to. These fragment structures with scores are written out to a SDF file (Fragments SDF), as they are helpful in identifying fragments with high NP-likeness. This workflow can be freely downloaded at http://www.myexperiment.org/workflows/2121.html.

Version 2.0 of CDK-Taverna provides two groups of workers that re-implement the work of *Ertl et al *to score small molecules for NP-likeness [14]. The workers in the Molecule Curation folder are dedicated to the pre-processing of chemical structures: The Molecule Connectivity Checker worker removes counter ions and disconnects fragments, the Remove Sugar Groups worker removes all sugar rings and linear sugars from structures and the Curate Strange Elements worker discards structures that are composed of elements other than non-metals. This set of curation workers finally creates scaffolds olds and sub structures. From these structures atom signatures [16] are generated using the Generate Atom Signatures worker and exploited as structural descriptors in charting the compound's region in the chemical structure space. The combined workflow of curation and atom signature generation workers is illustrated in Figure 4. Using this workflow, atom signatures can be generated for user-defined training (Natural products and synthetics) and testing (compound libraries) structural dataset. Workers of the Signature Scoring folder use atom signatures generated from compound libraries and rank them for NP-likeness based on the statistics suggested by *Ertl et al *[14]. This scoring workflow is illustrated in Figure 5. The whole package of workflows is available for free download at http://www.myexperiment.org/users/10069/packs. The curation and signature scoring workers may not only be applied in evaluating the NP-likeness of compound libraries but also in evaluating the metabolite-likeness of theoretical metabolites for predicting whole metabolomes. The latter application was the original purpose for the worker development and corresponding results will be presented in a subsequent publication.

### Clustering and machine learning applications

Unsupervised clustering tries to partition input data into a number of groups smaller than the number of data whereas supervised machine learning tries to construct model functions that map the input data onto their corresponding output data. If the output codes continuous quantities a regression task is defined. Alternatively the output may code classes so that a classification task is addressed. Molecular data sets for clustering consist of input vectors where each vector represents a molecular entity and consists of a set of molecular descriptors itself. Molecular data sets for machine learning add to each input vector a corresponding output vector with features to be learned - thus they consist of I/O pairs of input and output vectors.

The clustering and machine learning workers of CDK-Taverna 2.0 allow the use of distinct WEKA functionality. As far as clustering is concerned the ART-2a worker of version 1.0 is supplemented with five additional WEKA-based workers which offer

• Expectation Maximisation (EM): Expectation maximisation algorithm for iterative maximum likelihood estimation of cluster memberships [17].

• Farthest First: Heuristic 2-approximation algorithm for solving the k-center problem [18].

• Hierarchical Clusterer: Hierarchical clustering methods: The distance function and the linkage type are freely selectable [19].

• Simple KMeans: Simple k-means clustering algorithm [20].

• XMeans: Extended k-means clustering with an efficient estimation of the number of clusters [21].

Machine learning workers support the significance analysis of single components (i.e. features) of an input vector to obtain smaller inputs with a reduced set of components/features, the partitioning of machine learning data into training and test sets, the construction of input/output mapping model functions and model based predictions as well as result visualization. There is a total of six WEKA-based machine learning methods available: Two workers allow regression as well as classification procedures...

• Three-Layer Perceptron-Type Neural Networks: Neural network implementation using the backpropagation algorithm for weight optimisation [22].

• Support Vector Machines: Support Vector Machine implementation using the LibSVM library [23].

... two workers do only support regression...

• Multiple Linear Regression: Multiple linear regression algorithm.

• M5P regression trees: M5 regression algorithm for constructing tree-based linear models [24,25].

... and two workers are restricted to classification tasks:

• Naive Bayes: Bayesian classifier for the estimation of continuous variables [26].

• J46 C4.5 decision tree: Decision tree implementation based on the C4.5 classification algorithm [27].

**Figure 6:**
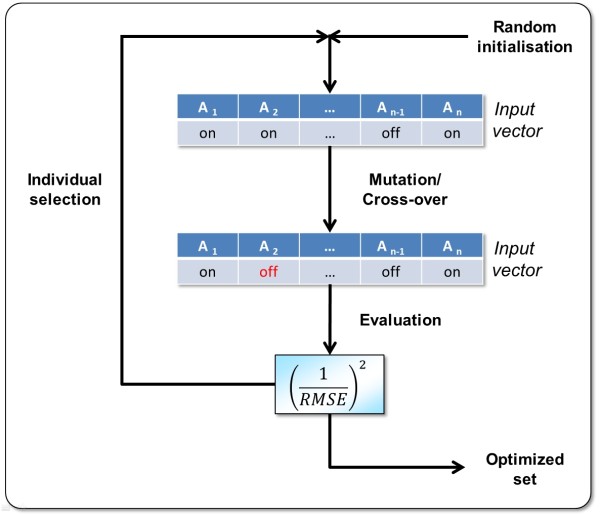
**Genetic algorithm for selection of an optimum reduced set of input vector components: The algorithm starts with a random population in which each chromosome consists of a random distribution of enabled/disabled (on/off) input vector components denoted *A*_1 _to *A_n _*(where the number of components with "on" status remains fixed during evolution)**. This distribution is changed by mutation and cross-over. The fitness of each chromosome is evaluated by the inverse square RMSE. The selection process for each generation is performed by Roulette wheel selection where chromosomes are inherited with probabilities that correspond to their particular fitness.

**Figure 7:**
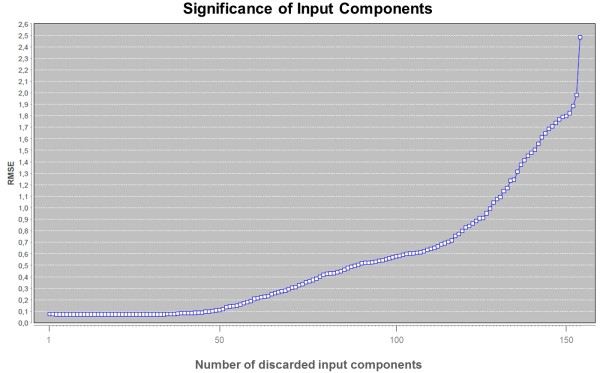
**"Leave-One-Out" analysis to estimate the significance of input vector components: The root mean square error (RMSE) rises with an increasing number of discarded components (i.e. a decreasing number of input vector components used for the machine filearning procedure)**. The relative RMSE shift from step to step may be correlated with the significance of the discarded component. In this case it is shown that the first fifty components do only have a negligible in influence on the machine learning result and thus may be excluded from further analysis.

**Figure 8:**
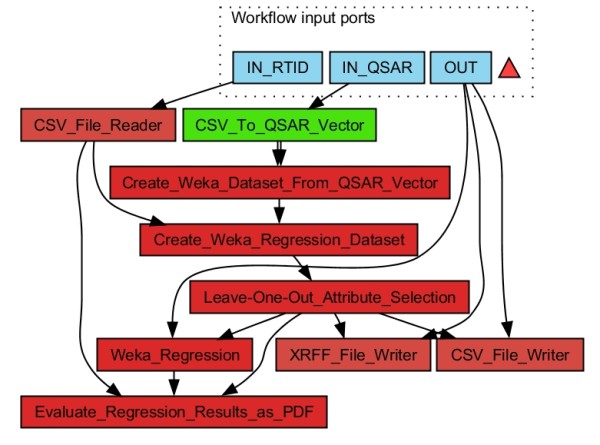
**Workflow for "Leave-One-Out" analysis: First a regression dataset is generated from a CSV file with UUID and molecular descriptor input data for each molecule (**IN QSAR**) and a CSV file containing the UUID of the molecule and the corresponding output (regression) value (**IN RTID**)**. Then the Leave-One-Out Attribute Selection worker evaluates the significance of the input components and generates a dataset for each evaluation step. Afterwards the composed datasets are coded as XRFF files. A CSV file with the sequence of discarded input vector components is generated. In addition the results are visualised with a PDF output file. Instead of the Leave-One-Out Attribute Selection worker a GA Attribute Selection worker may be used to determine a minimum molecular descriptor subset with maximum predictability. The results are stored in the output folder determined by the OUT input port.

For selection of an optimum reduced set of input vector components there are two workers available. The GA Attribute Selection worker generates an optimum reduced set of input components of predefined length (smaller than the full input vector length) on the basis of a genetic algorithm. The initial random population is refined by mutation and cross-over steps plus Roulette Wheel selection in each generation. A mutation switches an input component between an "on" or "off" state and a cross-over interchanges a random interval of "on/off" states between two randomly chosen chromosomes (where the number of attributes with "on" state remains fixed). As a fitness function the inverse square root mean squared error ${\left(\frac{1}{RMSE}\right)}^{2}$ is used - based on the complete dataset or using n-fold cross-validation. Figure 6 illustrates the procedure. The Leave-One-Out Attribute Selection worker uses a "Leave-One-Out" strategy for evaluating the significance of each input vector component [28]. In each iteration the single component is discarded that has the smallest in influence on the RMSE - up to a last "most significant" component. Figure 7 shows a result of a "leave-one-out" analysis and Figure 8 depicts the related workflow.

For training and test set partitioning the Split Dataset Into Train-/Testset worker is available which offers three strategies [28]:

• Random: Data are split randomly into a training and test set of defined sizes.

• Cluster Representatives: First the input data of the I/O pairs are clustered with the number of clusters to be equal to the number of training data by application of the Simple KMeans algorithm. Then a single input point of each cluster is chosen randomly as a representative and the corresponding I/O pair is inserted into the training set. The remaining I/O pairs are transferred to the test set.

• Single Global Max: Cluster representatives are evaluated in a first step. These representatives are then re ned by an iterative procedure that exchanges data between training and test set that belong to the same cluster. The latter constraint assures that the input data of training and test set have a similar spatial diversity. A single iteration determines the test set I/O pair with the largest deviation between data and model. This I/O pair is then transferred to the training set while the best predicted I/O pair of the same cluster in the training set is transferred to the test set in exchange. Oscillations during the refinement steps may be suppressed by blacklisting exchanged I/O pairs.

**Figure 9:**
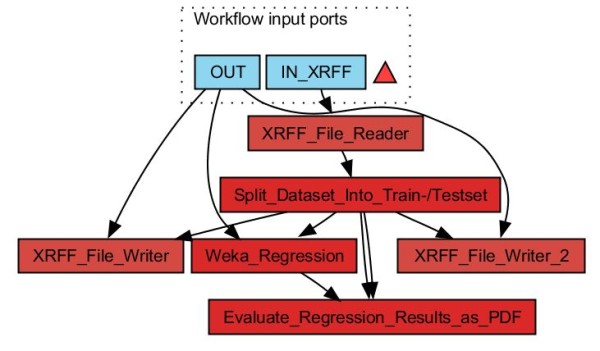
**Partitioning into training and test set: A regression dataset is split into a training and a test set which is performed by the **Split Dataset Into Train-/Testset. Then a regression model is created by the Weka Regression worker and evaluated by the Evaluate Regression Results as PDF which stores the results in a PDF file. The dataset is read from a XRFF file (IN XRFF). The generated test and training sets are coded as XRFF files and stored on hard disk. The OUT input port determines the result output folder.

**Figure 10:**
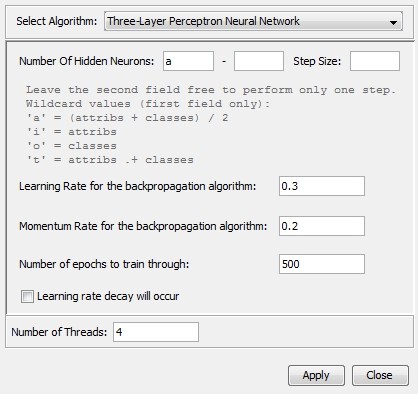
**Configuration panel for the Weka Regression worker: The configuration for a three-layer perceptron neural networks is selected**. Each machine learning method consists of a parameter panel for individual configuration.

**Figure 11:**
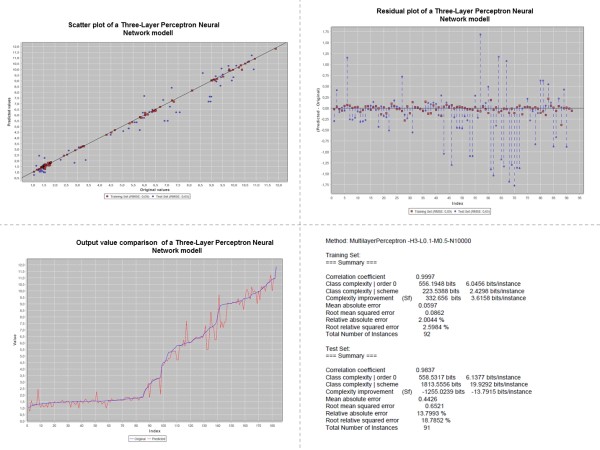
**Diagrams for machine learning results:** (upper left) Scatter plot with experimental versus predicted output values. (upper right) Residuals plot with differences between the predicted and experimental output values. (lower left) Experimental output data are plotted over corresponding sorted predicted output data. (lower right) Characteristic quantities of the predicted model.

Figure 9 shows a workflow using the Split Dataset Into Train-/Testset worker. The Weka Regression worker is used to build machine learning models which may be evaluated and visualized by the Evaluate Regression Results as PDF worker. The Weka Regression worker provides a configuration menu as shown in Figure 10. Classification workers may be used in an equivalent manner. Figure 11 depicts diagrams and output of a QSPR analysis to predict HPLC retention times for small molecules: The experimental dataset consists of 183 I/O pairs with a set of molecular descriptors for each small molecule as an input and the corresponding retention time as an output. The molecular descriptors were calculated with the QSAR Descriptor Threaded worker. Afterwards the GA Attribute Selection worker was used to determine an optimized minimum subset of 75 molecular descriptors (from an original 155) with maximum predictability. For machine learning a three-layer perceptron type neural network worker with three hidden neurons was used. The diagrams shown for the regression analysis are a scatter plot with experimental versus predicted output values and two kinds of residual plots. In addition characteristic quantities like the root mean squared error or the correlation coefficient are calculated for the generated model.

### CDK-Taverna 2.0 Wiki

Based on the free MediaWiki framework a Wiki was developed for the CDK-Taverna 2.0 project [29]. The web page provides general information about the project, documentation about available workers/workflows and on how to create them as well as about installation procedures. The Wiki can be found at http://cdk-taverna-2.ts-concepts.de/wiki/index.php?title=Main_Page.

## Conclusions

CDK-Taverna 2.0 provides an enhanced and matured free open cheminformatics workflow solution for the biosciences. It was successfully applied and tested in academic and industrial environments with data volumes of hundreds of thousands of small molecules. Combined with available workers and workflows from bioinformatics, image analysis or statistics CDK-Taverna supports the construction of complex systems biology oriented workflows for processing diverse sets of biological data.

## Competing interests

The authors declare that they have no competing interests.

## Authors' contributions

EW initiated the integration of Taverna and CDK and supported deployment and architecture. CS and AZ conceived the project and lead the further development. SN supported the reaction enumeration enhancements. KV provided workers for molecular fragmentation. AT did the majority of CDK-Taverna re-engineering and enhancements and developed the project to its current state. All co-authors contributed to the manuscript. All authors read and approved the final manuscript.
